# Cleavage and in vitro cultivation rates monitoring in culture media supplemented with energy sources, non-essential amino acids, and antioxidants in the buffalo embryos

**DOI:** 10.1186/s12917-024-04118-4

**Published:** 2024-11-18

**Authors:** Eman M Abu El-Naga, Montaser E. Ali, Rawda H. Ali, Heba F. Hozyen, Hassan A. Hussein

**Affiliations:** 1 Department of Theriogenology, Faculty of Veterinary Medicine, Aswan University, Aswan, Egypt; 2 Animal Production Department, Faculty of Agriculture, Assiut Branch, Al-Azhar University, Assiut, Egypt; 3 Department of Animal Production, Faculty of Agriculture, Assiut University, Assiut, 71526 Egypt; 4 Animal Reproduction and Artificial Insemination Department, Veterinary Research Institute, National Research Center, Giza, Egypt; 5 Physiology and Biochemistry Department, Faculty of Veterinary Medicine, Ain Shams University, Cairo Governorate, Egypt; 6 Department of Theriogenology, Faculty of Veterinary Medicine, Assiut University, Assiut, 71526 Egypt; 7 Department of Theriogenology, Faculty of Veterinary Medicine, Sphinx University, New Assiut, 71684 Egypt

**Keywords:** IVC, Energy sources, non-essential amino acids, Antioxidants, SOF, Ferticult, Buffalo embryo

## Abstract

The study was designed to monitor the cleavage rate (CR) and *in-vitro* cultivation rate (IVC) after addition of energy sources, non-essential amino acids, and antioxidants to the Synthetic oviductal fluid (SOF) and FertiCult. After *in-vitro* maturation and *in-vitro* fertilization, presumptive zygotes were cultured in one of two culture media: FertiCult media and SOF medium, supplemented with pyruvate, glucose, and sodium lactate as energy sources, as well as 10, 20, 250, 500, and 750 mg non-essential amino acids, and antioxidants. All stages of cleavage rate (CR), and *in-vitro* cultivation rate (IVC) of embryonic development including morula stage (MOR) and blastocyst (BLAS) have been assessed. The findings revealed that there were no significant differences in the CR between the control and other treated groups with sources of energy when added to SOF media (*P* > 0.05), while there were significant differences (*P* < 0.05) in the IVC of embryonic development between groups (The percentages of MOR stage in the control, pyruvate, glucose and mixture of source of energy (MIX) were at 50%, 62.5%, 60%, and 63.6%, respectively). The highest percentage of the BLAS was recorded after SOF supplementation with glucose (40%). Similarly, there were no significant differences (*P* > 0.05) in the CR between control and FertiCult supplemented with sources of energy, while the IVC stages increased significantly (*P* < 0.05) in the FertiCult media supplemented with glucose, pyruvate, sodium lactate, and MIX. The percentages of the MOR stage in the control, pyruvate, glucose and mix media were at 50%, 55.6%, 55.6%, 54.5%, 57.1% respectively. The lowest percentage of the BLAS was recorded after FertiCult supplementation with pyruvate (11.1%). Replenishing the SOF maturation media with 20 mg of non-essential amino acids significantly (*P* < 0.05) enhanced the MOR stage (100%). There was also an improvement in the development of BLAS stage, where it reached 31.2% and 47.4% in the SOF maturation media supplemented with 10, and 750 mg non-essential amino acids, respectively. There were no significant differences (*P* > 0.05) in neither CR nor IVC between control and FertiCult supplemented with antioxidants. There were significant differences (*P* < 0.05) in the MOR stages (control, 42.9% & treated, 57.9%) and BLAS stages (control, 21.4% & treated, 42.1%) in antioxidant supplemented SOF maturation media compared to control. In conclusion, supplementation of SOF cultivation medium with energy sources, 20 mg of non-essential amino acids and antioxidant addition may improve the cleavage rate (CR) and in vitro cultivation rate (IVC) of buffalos’ embryonic development.

## Introduction

One of the earliest forms of assisted reproductive technology employed in the livestock industry is the use of artificial insemination, in which the ejaculate of a single, genetically superior bull was used after semen processing and extension to many doses to inseminate numerous cows [1]. However, the improvement in dairy industry remained limited as genetically superior cows were restricted to a single calf per breeding season [2].

Achieving the maximum number of offspring born from a dam is critical for increasing the pace of genetic improvement throughout the cattle flocks [3]. This task can be accomplished using in-vitro cultivation (IVC) technology [4]. Furthermore, various culture media for IVC of buffalo embryos have been studied. For example, SOFs are synthetic oviduct fluids that were manufactured using a combination of oviduct fluids from bovines [5, 6], and FertiCult is also a salt solution chemically balanced with gentamycin, used in embryo replacement, swim-up technique, and washing of the sperm during sperm selection [6, 7].

Adenosine triphosphate (ATP) formation in buffalo oocyte and embryo culture has been impacted by pyruvate, glucose, and amino acids [8]. Therefore, its presence in the cultivation media is considered necessary. It has been observed that adding glucose to the culture medium is crucial for the development of buffalo oocytes and embryos [9]. However, pyruvate is a constant source of energy in cultured oocyte and embryos, as in the early embryonic stages sodium pyruvate is the preferred substrate for tricarboxylic acid [10–12]. Likewise, Takahashi and First [13] reported that in the absence of glucose, lactate and pyruvate can promote bovine embryo growth.

The presence of non-essential amino acids permits in vitro oocytes and embryos to withstand high physiological osmolality; they serve as a protective shield, allowing the organism to maintain its form and thrive in high osmotic environments [14, 15].

The oxidative stress caused by poor culture conditions slowly compromises in vitro embryo development [16], it leads to disruption of the functional integrity of cells by reactive oxygen species [17, 18]. Because buffalo oocytes and embryos have high lipid content, they are probably more susceptible to oxidative injury [19].

Marin et al. [9] reported that, it has been challenging to provide appropriate culture media for buffalo embryos due to a lack of knowledge on their metabolic and biochemical requirements. Also, there is also little information available on the addition of non-essential amino acids to buffalo embryo cultivation media [20]. As a result, the aim of this investigation was to monitor the cleavage rate (CR) and in vitro cultivation rate (IVC) of buffalo embryos by supplementing SOF and FertiCult media with energy sources (pyruvate, glucose, and sodium lactate), non-essential amino acids, and antioxidants.

## Materials and methods

### Collection of ovaries

Within thirty minutes of slaughtering of adult buffalo-cows in a local abattoir in Aswan, the ovaries were collected. The ovaries were maintained in a thermal flask filled with normal physiological saline (0.9% NaCl) supplemented with 50 µg/mL of streptomycin and 400 IU/mL penicillin, and kept at 37 °C, then sent to the laboratory of Aswan University, Department of Theriogenology, Faculty of Veterinary Medicine within two hrs [21]. . The saline temperature was maintained at a specific level during the transportation of ovaries to the laboratory to ensure the viability of the oocytes within the ovaries.

### Semen used for IVF

Frozen semen straws from fertile and valuable buffalo bulls previously checked and stored in liquid nitrogen (-196 °C) were procured from the Artificial Aswan Veterinary Directorate, which received straws from the General Authority for Veterinary Services-Egypt’s Abbasiya Center. Sperm TALP (SP-TALP) was used to select spermatozoa prior to capacitation using the swim up procedure. The spermatozoa were cleaned twice with centrifugation (x500g/10 min). TALP Medium used for sperm capacitation, and in vitro fertilization [22].

### Collection media

The cumulus oocyte complexes (COCs) collection was carried out using collection media medium-199, which contained L-glutamine, trace amount of antibiotics (streptomycin 100 mg/ml, penicillin 100.000 IU/ml), 25 mM HEPES, Earle’s salts, and 3% (v/v) heat-inactivated calf serum (heat-treated at 56 °C for 30 min). It was refrigerated at 5 °C until needed.

### Maturation media

There were two types of media used to assess cleavage, and in vitro cultivation rate of embryonic development: Synthetic oviductal fluid (SOF [23]), , which was made fresh in the lab and refrigerated at 5 °C until it was needed. 10% fetal calf serum (FCS) along with gentamycin was added to SOF as a supplement. The second is a liquid medium known as FertiCult (FertiPro N.V., Belgium), which is an aqueous solution containing HEPES, bicarbonate, physiologic salts, glucose, lactate, pyruvate and 2.5 IU/ml Heparin. FertiCult was kept in a refrigerator at 5 °C until it is needed.

### Preparation of media

Before culture, all the media utilized in this experiment had been filtrated using a 0.2 μm Syringe filter (Millipore, USA) and incubated for at least two hours in a 95% humidified atmosphere with 5% CO_2_ at 38 °C.

### Oocyte recovery and in vitro maturation

Ovaries were washed in a water bath for oocyte collection after being repeatedly cleaned 0.9% NaCl solution at 37 °C. With sterile paper towels ovaries were dried, COCs were extracted from visible follicles using a 18 gauge needle attached to 10 mL syringe in medium-199 which contains Earle’s salts, L-glutamine, HEPES, heat-treated calf serum, and antibiotics was added [24]. Number of 5 to 15 COCs have been pooled in 35-mm Petri plates during 24 h at 5% CO_2_, 38.5 °C, and 90–95% humidity ratios in 50–100 µL droplets of the filtrated IVM medium (pH 7.4) overlaid with sterilized mineral oil [25].

COCs with more than five compact cumulus cell layers, homogenous cytoplasm, and intact zona pellucida were used for in IVM after maturation the oocytes were used for IVF [26]. In accordance with Hufana-Duran’s [27] protocol, after maturation, oocytes were extracted from cumulus cells using 0.1% hyaluronidase, fixed in 1:3 aceto-ethanol, dehydrated, stained with 1% aceto-orcein, and de-stained with aceto-glycerol. Furthermore recovered oocytes were categorized based on their quality into three groups: denuded oocytes with uneven ooplasm, fair oocytes with homogeneous ooplasm surrounded by 1–3 layers of cumulus cells, and good oocytes with homogenous granular ooplasm surrounded by more than three compact layers of cumulus cells [28].

The cellular maturation of oocytes was assessed using a compound microscope set at 200 X – 400 X to determine the maturation rate.

### In vitro fertilization

Matured oocytes were partially denuded from the surrounding cumulus cells to allow easy penetration of the sperm cells. They were washed twice in pre-warmed IVF medium (F- TALP medium). A total of 15–20 matured oocytes were placed in each well of the culture dish containing 50 µl of fertilization medium to which 20 µl of sperm suspension was added (the sperm concentration in the sperm suspension was 2 × 10^6^ sperm cells/mL). A layer of 200 µl of sterile liquid light mineral oil was placed to cover the sperm-oocytes mixture then incubated for 18–20 h at 38.5ºC under 5% CO2 in humidified air [29].

### In vitro embryo culture

The presumed embryos were washed four times then cultured in culture media SOF, and FertiCult. Morula production rate was investigated from 94 to 96 h post-insemination for cell stages 8–16. The blastocysts formed and began to show one day later, 120 h after fertilization. To reduce the negative effect of the waste products produced by the embryos, a portion of the SOF-medium was replaced with a fresh one 2–3 times (pre-incubated for at least 2 h) prior to investigation [30]. Under a microscope, the first and second polar bodies were seen after the oocytes and sperm were co-incubated for 18 to 20 h. Following the gametes co-incubation period, a portion of the inseminated oocytes were separated from the cumulus cells that were attached. They were then preserved in a solution of acetic acid and ethanol (1:3), stained with 1% aceto-orcein stain, and observed using a ×400 phase-contrast microscope to determine the in vitro fertilization rate [22]. After cultivation on the following media with different supplementations, the cleavage rate (CR), and in vitro cultivation rate (IVC) of embryonic development was assessed using the same compound microscope set [31].

### Experimental design and addition of energy source, non-essential amino acids, antioxidants

**Fig. 1:**
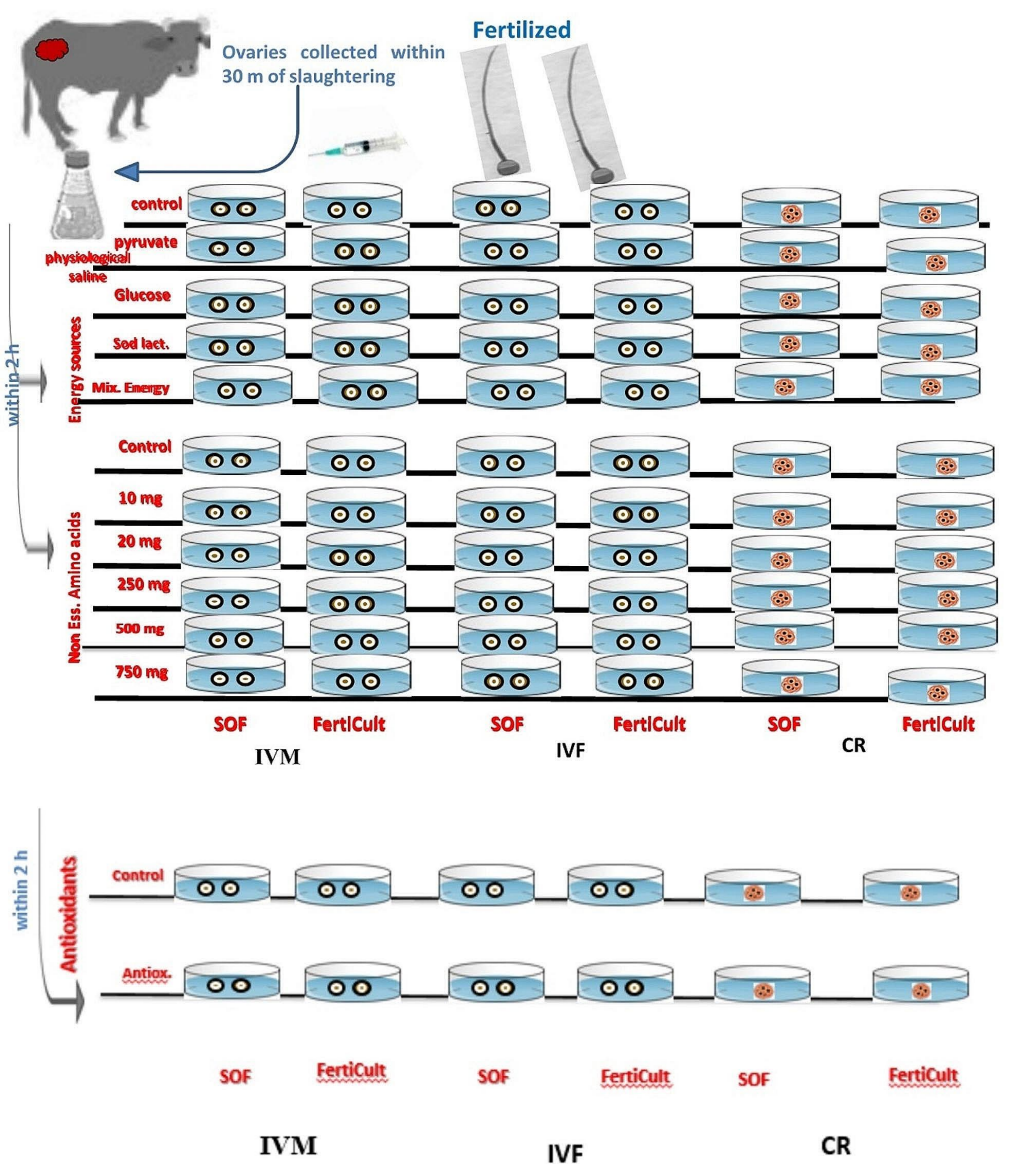
Illustrating diagram showing the experimental design and subgrouping; IVM: In vitro maturation of oocytes, IVF: in vitro fertilization; CR: Cleavage rate then in vitro cultivation (IVC, Morula and blastocyst)

- The experimental design was illustrated in diagram (Fig. 1). One of four distinct energy sources; glucose 5.6 mM [32], pyruvate (0.17 mM) [33], sodium Lactate (2.73 mM) [33], and mix of energy sources was supplemented to SOF, and FertiCult media during cleavage, and in-vitro cultivation of embryos (morula and blastocyst stage).
- One of the six concentrations of non-essential amino acids; 0 (control), 10, 20, 250, 500 and 750 mg was supplemented to SOF, and FertiCult during cleavage rate, and in-vitro cultivation (morula and blastosyste stage) of embryonic development.
- The presumptive embryos in the three control groups were without antioxidants addition, without energy sources addition, and without essential amino acids addition, and they were subdivided further into two groups (control groups for both SOF and FertiCult).

### Statistical analysis

Each experiment was at least three times replicated. Non-parametric data were reported as percentages of cleavage rate, and in vitro embryonic development (morula and blastocyst stages) was determined using the Chi-square trend test. The statistical significance of the difference was determined using the chi-square or 2 test in SPSS version 16. Values with different letters differ significantly among treatments and when p-value was < 0.05, all differences were considered significant.

## Results

Tables (1&2), displays the impacts of energy sources: pyruvate, glucose, sodium lactate, and mix supplementation on the SOF and FertiCult media on cleavage rate (CR) and in vitro cultivation rate (IVC) and embryonic development. The findings revealed that there were no significant differences in the CR between the control and other treated groups with sources of energy when added to SOF media (*P* > 0.05), while there were significant differences (*P* < 0.05) in the IVC: the percentages of MOR stage in the control, pyruvate, glucose and mixture of source of energy (MIX) were at 50%, 62.5%, 60%, and 63.6%, respectively, where the highest MOR percentage recorded in MIX. The highest percentage of the BLAS was recorded after SOF supplementation with glucose (40%) (Table 1). Similarly, there were no significant differences (*P* > 0.05) in the CR among the control and supplemented with sources of energy FertiCult media. while the IVC stages increased significantly (*P* < 0.05) in the FertiCult media supplemented with glucose, pyruvate, sodium lactate, and MIX. The percentages of the MOR stage in the control, pyruvate, glucose, sodium lactate and mix media were at 50%, 55.6%, 55.6%, 54.5%, 57.1% respectively. The lowest percentage of the BLAS was recorded after FertiCult supplementation with pyruvate (11.1%) (Table 2).

**Table 1:** Cleavage rate (CR) and in-vitro cultivation rate (IVC) of embryonic development in the SOF media supplemented with sources of energy

Items	CR			IVC				
				MOR		BLAS		
	*F	%	*N	F	%	F	%	*N*
Control	8	26.7	30	4	50.0	-	-	8
Pyruvate	8	33.3	24	5	62.5	-	-	8
Glucose	10	41.7	24	6	60.0	4	40.0	10
Sodium lactate	11	40.7	27	7	63.6	3	27.3	11
MIX	11	50.0	22	7	63.6	4	36.4	11
Total number	127			48				
**Chi (P-value)**	3.43 (0.489)^NS^			17.382 (0.026)^*^				

*F: the number of cells added to the media, *N: replicate observations of cells that grew, Superscript NS: non-significant difference, *: significant difference (*P* < 0.05)

**Table 2:** Cleavage rate (CR), and in-vitro cultivation rate (IVC) of embryonic development in the FertiCult media supplemented with sources of energy

Treatments	CR			IVC				
				MOR		BLAS		
	*F	%	*N	F	%	F	%	*N*
Control	6	27.3	22	3	50.0	-		6
Pyruvate	9	39.1	23	5	55.6	1	11.1	9
Glucose	18	47.4	38	10	55.6	8	44.4	18
Sodium lactate	11	34.4	32	6	54.5	4	36.4	11
MIX	14	48.3	29	8	57.1	5	35.7	14
Total number	144			58				
**Chi (P-value)**	3.59 (0.465)^NS^			15.6 (0.049)^*^				

*F: the number of cells added to the media, *N: replicate observations of cells that grew, Superscript NS: non-significant difference, *: significant difference (*P* < 0.05)

Tables (3 & 4), show the effects of supplementing the SOF and FertiCult media with 10, 20, 250, 500, and 750 mg non-essential amino acids on cleavage rate (CR) and in vitro cultivation rate (IVC) embryonic development. Replenishing the SOF maturation media with 20 mg non-essential amino acids significantly (*P* < 0.05) enhanced the MOR stage, but its cells did not develop the BLAS form. Furthermore, there was an improvement that reached 31.2% and 47.4 of the MOR cells that formed the BLAS stage in the SOF maturation media with 10 mg and 750 mg non-essential amino acids, respectively (Table 3). While there were no significant differences in both CR and IVC of embryonic development in the FertiCult media supplemented with non-essential amino acids (Table 4).

**Table 3:** Cleavage rate (CR), and in-vitro cultivation rate (IVC) of embryonic development in the SOF media supplemented 10, 20, 250, 500, and 750 mg non-essential amino acids

Treatments	CR			IVC				
				MOR		BLAS		
	*F	%	*N	F	%	F	%	*N*
Control	9	33.3	27	4	44.4	-	-	9
10 mg	16	51.6	31	8	50.0	5	31.2	16
20 mg	6	31.6	19	6	100.0	-	-	6
250 mg	10	29.4	34	6	60.0	2	20.0	10
500 mg	12	32.4	37	8	66.7	3	25.0	12
750 mg	19	51.4	37	9	47.4	8	42.1	19
Total number	185			72				
**Chi (P-value)**	7.24 (0.203)^NS^			18.524 (0.047)^*^				

*F: the number of cells added to the media, *N: replicate observations of cells that grew, Superscript NS: non-significant difference, *: significant difference (*P* < 0.05)

**Table 4:** Cleavage rate (CR), and in-vitro cultivation rate (IVC) of embryonic development in the FertiCult media supplemented 10, 20, 250, 500, and 750 mg non-essential amino acids

Treatments	CR			IVC				
				MOR		BLAS		
	F	%	*N*	F	%	F	%	*N*
Control	6	27.3	22	2	33.3	-	-	6
10 mg	13	43.3	30	8	61.5	4	30.8	13
20 mg	6	20.0	30	3	50.0	1	16.7	6
250 mg	8	29.6	27	4	50.0	2	25.0	8
500 mg	9	30.0	30	5	55.6	2	22.2	9
750 mg	10	35.7	28	6	60.0	2	21.2	10
Total number	167			52				
**Chi (P-value)**	4.29 (0.203)^NS^			8.706 (0.56)^NS^				

*F: the number of cells added to the media, *N: replicate observations of cells that grew, Superscript NS: non-significant difference, *: significant difference (*P* < 0.05)

The effects of antioxidants supplementation to the SOF and FertiCult media on the cleavage rate (CR) and in vitro cultivation rate (IVC) of embryonic development are displayed in Tables (5 & 6). There were significant differences (*P* < 0.05) in the antioxidant supplemented SOF maturation media in the MOR and BLAS stages compared to control, while there were no significant differences in the CR (Table 5). On other hand, there were no significant differences in the CR and IVC in the antioxidant supplemented FertiCult maturation media (Table 6).

**Table 5:** Cleavage rate (CR), and in-vitro cultivation rate (IVC) of embryonic development in the SOF media supplemented antioxidants

Treatments	CR			IVC				
				MOR		BLAS		
	F	%	*N*	F	%	F	%	*N*
Control	14	41.2	34	6	42.9	3	21.4	14
Antioxidant	19	63.3	30	11	57.9	8	42.1	19
Total number	64			33				
**Chi (P-value)**	3.133 (0.077)^NS^			8.173 (0.017)^*^				

*F: the number of cells added to the media, *N: replicate observations of cells that grew, Superscript NS: non-significant difference, *: significant difference (*P* < 0.05)

**Table 6:** Cleavage rate (CR) and in-vitro cultivation rate (IVC) of embryonic development in the FertiCult media supplemented antioxidants

Treatments	CR			IVC				
				MOR		BLAS		
	F	%	*N*	F	%	F	%	*N*
Control	12	41.4	29	8	66.7	4	33.3	12
Antioxidant	21	45.7	46	12	57.1	8	38.1	21
Total number	75			33				
**Chi (P-value)**	0.132 (0.717)^NS^			0.733 (0.693)^NS^				

*F: the number of cells added to the media, *N: replicate observations of cells that grew, Superscript NS: non-significant difference, *: significant difference (*P* < 0.05)

## Discussion

The present study was undertaken to monitor the cleavage rate (CR) and *in-vitro* cultivation rate (IVC) of in vitro produced buffalo embryo after addition of energy sources, non-essential amino acids, and antioxidants to the cultivation media SOF and FertiCult. Generally, energy sources play an important role in affecting cell metabolism at various stages [34]. According to reports, the metabolism of embryos is biphasic; during the period of embryonic activation, energy metabolism shifts from using the Krebs cycle and oxidative phosphorylation to use lactate and pyruvate to primarily use glucose through the Embden-Meyerhof pathway [35, 36]. At every stage of the IVF process beginning with oocytes maturation till the resulted embryonic development, glucose was noticed to be critical [23]. The presented results demonstrated that glucose, pyruvate, and sodium lactate as energy sources boost the cleavage rate (CR) and in-vitro cultivation rate (IVC) of embryonic growth in SOF media. This lends support to the idea that energy sources have a functional role in oocyte and follicle maturation [37]. Even though the oocytes matured without glucose, they were still able to cleave, go through the earliest phases of cleavage, and perhaps even pass through the maternal to zygotic transition (MZT) stage. However, a marked greater proportion of these oocytes were noticed to be arrested at the morula stage [32]. It was found that after supplementation of the cultivation media with the ideal glucose concentration (5.6 mM) in both the IVC-I and IVC-II stages, only around 9% of these embryos (which were produced from oocytes that had been starved of glucose during IVM) could develop to the blastocyst stage. These embryos may have been produced from higher-quality oocytes that were either taken from follicles with rich microenvironments [38] or have reserves of energy that showed immature oocytes included different biochemical compositions. The in vitro maturation of pig oocytes and embryos is dependent on reserve triglycerides in the oocytes [39].

According to data in Table (1) of the current investigation, morula cells atimulated with glucose in SOF medium, while that in FertiCult medium failed to develop and didn’t reach the blastocyst stage, which in consistent with previously published data [40]. There are reports that, in a few species, glucose may be detrimental to the development of embryos in vitro. In hamster, it was found that high glucose level at two cell stage in vitro; could stop the further growth to four cell stage [41]. However, attainment of the highest blastocyst percentage in the current study was recorded after supplementation of cultivation media SOF and FertiCult with 5.7 mM glucose (Tables 1 and 2), these findings agreed with those reported before [32, 42]. Additionally, it was reported that embryos utilize the endogenous nutrients while they are in physiological condition [43]. It is clear from the obtained results that when the cultivation media supplemented with non-ideal concentration of source of energy showed lesser number of cleaved embryos reaching blastocyst stage with significantly higher percentage blocked at morula stage as it showed in Table (1) in case of control and pyruvate and in Table (2) in case of control. It was reported that pyruvate and lactate are very important energy supplements in porcine, especially during early embryonic development in vitro, while blastocyst formation requires much energy due to increased protein synthesis [44] and to increased activity of Na+/K+-ATPase [45]. It was observed in the present study that blastocyst rate varied significantly under different energy source levels supplemented to SOF and FertiCult. Previously it was reported that the high glucose concentration (10 mM) had deleterious during oocyte maturation and early embryonic development [32], unfortunately in the current work, addition of different concentrations of energy source to cultivation media weren’t investigated.

The current results showed that the replenishing the SOF media with non-essential amino acids improved the MOR stage substantially. However, essential and non-essential amino acids are both necessary for maintaining cell viability *in-vitro* [46]. There hasn’t been much research on the needs for amino acids (AAs) at different stages of the embryo, even though giving a variety of amino acids aids in preimplantation embryo development. Apart from the fact that glycine and alanine are the two main AAs that must be consumed at higher quantities for IVC, methionine is also becoming increasingly important because of how it interacts with folate [47]. The embryo consumes AAs and carboxylic acids as energy sources before the embryonic genome is expressed [48]. Furthermore, it has been established that certain AAs can serve as precursor molecules for biosynthesis [49], osmolytes [50], internal pH buffers [51], antioxidants [52], and chelators for heavy metals [53]. Glutamate and the seven non-essential AAs promote the development of the early cleavage embryo [54]. On the other hand, it was observed that blastocyst viability and development are inhibited if the thirteen essential AAs are given early [55]. While the non-essential AAs and glutamine stimulate the throphectoderm and hatching from the zona pellucida, both types of AAs operate as stimulants to the inner cell mass of blastocysts in the post-compaction stage [56, 57]. So, protein synthesis needs to happen before this stage for bovine embryos to develop correctly into blastocysts [58]. Moreover, compared to media designated without amino acids, the addition of amino acids to oocyte maturation media has been linked to improved growth frequencies, higher levels of maternal oocyte mRNA, and larger numbers of blastocyst cells [59]. In the current investigation, non-essential amino acid-supplemented FertiCult medium did not significantly alter the CR or IVC of embryonic development, this result may be due FertiCult media contains amino acids or may be due to AAs in culture media also spontaneously break down, ammonium is released into the medium, with a time dependent concentration. Ammonium decreases the viability of embryos and is harmful to them [60]. To overcome the accumulation of the ammonium AAs should added in a two-step (sequential) culture media [55]. Furthermore, it should be mentioned that enough amounts of sulfur-containing amino acids in culture media are necessary to reduce apoptosis, which results in monozygote twinning [61].

Reactive oxygen and nitrogen species (RONS), which are naturally produced in an oxidative environment and have the potential to harm embryo development, have been the subject of much research on in vitro fertilization (IVF). The most used marker of choice in assessing the redox status is the ratio of reduced glutathione (GSH) to oxidised glutathione (GSSG). The endoplasmic reticulum (ER) is substantially less reducing than the cytoplasm, which is a strong reducing environment based on ratios of GSH/GSSG [62]. Although reactive oxygen species (ROS) in cells are beneficial to embryogenesis and tissue regeneration, excessive amounts can oxidize cellular molecules, jeopardizing viability and leading to apoptosis, lipid peroxidation, and mitochondrial damage [63]. An increase in ROS species may occur during IVM as a result of oxygen tension in the culture medium used [64]. In this study, there were significant differences in the antioxidant system supplemented with SOF maturation media in the MOR and BLAS stages compared to the control. This leads to improving oocyte maturation, and embryo development. On other hand, there were no significant differences in the CR and IVC in the antioxidant system supplemented with FertiCult maturation media. Over the past 20 years, numerous studies have been carried out to reduce oxidative stress during IVC. Antioxidants combined with hypoxic (i.e., 5–10% O_2_) embryo culture conditions partially offset the antioxidant benefits of co-culture cells. Controversial conclusions have come from studies incorporating antioxidants in both high and low oxygen tension circumstances. In the last decade, the impact of GSH, β-ME, and cysteine as antioxidant on embryo development using a pig model was investigated. Treatment groups contained more developing embryos than the control group [65], however because high O_2_ culture conditions (20% O_2_ and 5% CO_2_) were utilised, larger concentrations of these antioxidants may have enhanced embryo production. In contrast, at reduced oxygen tension (5% O_2_), guaiazulene (a component of several chamomile species with antioxidant properties) had no beneficial effect on embryonic development [67]. Although the difference in media used and animal species included, these findings agreed with those recorded in our study where addition of antioxidants to SOF cultivation medium has beneficial effect on the embryonic development, while no significant changes were recorded after its supplementation to FertiCult medium during IVEP of buffaloes.

## Conclusions

Based on our findings, it was concluded that a mixture of energy sources specially glucose and antioxidant addition markedly improved the in vitro cultivation rate (IVC) of the embryonic development in SOF medium. The CR not improved in both SOF and FertiCult media supplemented with source of energy. The supplementation of non-essential amino acids to the cultivation media SOF/FertiCult was very beneficial for the morula stage and blastocyst development. The existing data will be useful in establishing a successful buffalo IVF technique.

## Data Availability

The corresponding author can provide the study’s datasets upon reasonable request.

## Declarations

### Study approval and animal Accordance Statement

This study was conducted in accordance with Institutional and National Guidelines for the care and use of animals were followed according to the OIE standards, the Ethical Committee of the Faculty of Veterinary Medicine, with the permission number (06/2023/0095).
